# Functional Characterization of *tetR* in Tetracycline Resistance of *Aeromonas hydrophila*

**DOI:** 10.3390/vetsci13060577

**Published:** 2026-06-12

**Authors:** Nannan Shen, Ting Qin, Bingwen Xi, Kai Chen, Yifan Lu, Jun Xie

**Affiliations:** 1 Wuxi Fisheries College, Nanjing Agricultural University, Wuxi 214081, China; 2023113017@stu.njau.edu.cn (N.S.); 2024113020@stu.njau.edu.cn (Y.L.); 2 Key Laboratory of Aquatic Animal Nutrition and Health, Freshwater Fisheries Research Center, Chinese Academy of Fishery Sciences, Wuxi 214081, China; chenk@ffrc.cn (K.C.); xiej@ffrc.cn (J.X.)

**Keywords:** *Aeromonas hydrophila*, tetracycline resistance, TetR, efflux pump, transcriptome analysis

## Abstract

This study aimed to investigate the molecular basis of high-level tetracycline resistance to tetracycline antibiotics in *Aeromonas hydrophila* isolated from fish farming. Comparative genomic analysis of the tetracycline-sensitive strain NJ-35 and the tetracycline-resistant strain AH823 revealed that the tetracycline repressor gene *tetR* in AH823 had undergone base mutations, resulting in premature translational termination. The *tetR* gene in NJ-35 was inhibited using a plasmid-based antisense RNA strategy, and the knockdown efficiency was confirmed by RT-qPCR. The resulting *tetR* antisense RNA-expressing strain, AHtetR-as, exhibited significantly increased resistance to tetracycline antibiotics (minocycline, tetracycline, and doxycycline), but did not affect biofilm formation or hemolysis. Moreover, *tetR* knockdown in NJ-35 was associated with increased efflux activity and reduced intracellular doxycycline accumulation. Transcriptomic analysis revealed that genes encoding the 30S ribosomal subunit proteins showed a differential expression pattern, with *rpsO* upregulated and *rpsD* and *rpsP* downregulated. These findings suggest that *tetR* contributes to tetracycline resistance in *A. hydrophila* and is associated with broad transcriptional changes related to cellular transport and ribosomal function.

## Simple Summary

This study successfully constructed a stable plasmid-based antisense knockdown strain targeting *tetR* (designated AHtetR-as) of tetracycline-susceptible *Aeromonas hydrophila* and provided preliminary evidence that *tetR* is associated with tetracycline resistance. The knockdown strain AHtetR-as exhibited significantly increased resistance to tetracycline antibiotics. Moreover, *tetR* knockdown in NJ-35 was associated with increased efflux-related phenotypes and reduced intracellular doxycycline accumulation. Transcriptomic profiling further revealed that *tetR* knockdown was associated with changes in energy metabolism and altered expression of 30S ribosomal subunit-related genes. This study suggests that *tetR* is involved in tetracycline resistance, through possible transcriptional changes in genes related to energy metabolism and ribosomal function.

## 1. Introduction

*Aeromonas hydrophila*, a Gram-negative short bacillus, has emerged as one of the primary pathogenic bacteria in aquaculture [1]. It can induce bacterial septicemia, enteritis, and red-skin disease in a wide range of commercially important aquatic animals [2], such as *Carassius auratus* [3], *Ctenopharyngodon idellus* [4], *Cyprinus carpio* L. [5], and *Piaractus mesopotamicus* [6]. Characterized by a high outbreak frequency, an elevated infection rate, and a mortality rate of up to 100%, this pathogen inflicts substantial economic losses on the global aquaculture industry [7,8].

Tetracyclines, a class of vital broad-spectrum antibacterial agents, play an irreplaceable role in aquaculture and have become one of the most widely used antibiotic categories worldwide since their first discovery in the 1940s [9]. However, antimicrobial resistance (AMR) has rapidly developed as a global public health threat, driven by the indiscriminate use of antibiotics and the growing prevalence of antibiotic resistance genes (ARGs) [10,11]. *A. hydrophila* has been demonstrated to harbor multiple ARGs, including those conferring resistance to *β*-lactamases, quinolones, and tetracyclines [12]. At present, three core mechanisms of bacterial tetracycline resistance have been identified [13]: drug efflux pumps (e.g., Tet(A) and Tet(B)), ribosomal protection proteins (RPPs) (e.g., TetM and TetO), and enzymatic inactivation (e.g., Tet(X)) [14,15]. All three mechanisms have been reported in *Aeromonas* spp., and diverse *tet* genes including *tet*(A), *tet*(E), *tetM*, and *tet*(X) have been detected in *A. hydrophila*, frequently with multiple variants coexisting within a single strain [16,17]. *Aeromonas* spp. can acquire and disseminate mobile genetic elements such as plasmids, integrons, and transposons, which play a central role in the horizontal transfer of tetracycline resistance determinants across species and between aquaculture and clinical environments [18,19]. Acquired tetracycline-resistant *Aeromonas* spp. raises the issue of the One Health concept, which involves transmission of resistant pathogens to animals and humans who share an aquatic source through the food chain or direct contact [16,17]. Additionally, transcriptomic analyses in *A. hydrophila* have associated oxytetracycline resistance with broad efflux and metabolic gene remodeling [20], and TetR-family regulators extend beyond efflux control to govern metabolism and virulence [21], raising the possibility of pleiotropic effects from *tetR* mutations.

The canonical tetracycline resistance mechanism has been well characterized in model bacteria such as *Escherichia coli*. Briefly, the tetracycline–Mg^2+^ complex binds to TetR, inducing a conformational change that reduces its affinity for the *tet* operator and derepresses expression of Tet(A) efflux pump genes [22,23]. However, the regulatory complexity underlying tetracycline resistance in *A. hydrophila* remains insufficiently understood. In particular, the functional consequences of mutations in regulatory genes such as *tetR* and their contribution to high-level resistance in aquaculture-associated strains have not been fully elucidated.

In this study, we performed tetracycline resistance profiling on a collection of *A. hydrophila* isolates preserved in our laboratory for nearly a decade, and identified a clinical strain AH823 exhibiting high-level resistance to tetracyclines, with particularly high resistance to doxycycline. Genomic comparison between tetracycline-resistant *A. hydrophila* AH823 and susceptible strain NJ-35 (GenBank accession NO. NZ_CP006870.1) revealed a premature stop codon within the coding region of *tetR*, which is predicted to truncate the TetR protein and potentially impair its regulatory function. Based on the canonical model described above, a loss-of-function mutation in *tetR* would be expected to cause constitutive expression of *tet*(A) and increased efflux, making it the most parsimonious candidate to explain the high-level resistance observed in AH823. Nevertheless, the functional consequences of *tetR* mutations in aquatic *A. hydrophila* have not been experimentally defined, and the specific role of *tetR* in acquired tetracycline resistance of *A. hydrophila* remains undefined.

To address this knowledge gap, we employed tetracycline-sensitive *A. hydrophila* NJ-35 to construct a stable *tetR* antisense RNA-expressing strain, AHtetR-as, which exhibited increased tetracycline resistance. Subsequently, the key biological traits of AHtetR-as relative to NJ-35 were analyzed, including growth, hemolytic activity, biofilm formation, rhodamine B accumulation and membrane potential (Δ*Ψ*). Furthermore, transcriptomic profiling was integrated to elucidate the underlying mechanisms of the acquired tetracycline resistance in *A. hydrophila*. Overall, our findings demonstrate that *tetR* is involved in mediating acquired tetracycline resistance in *A. hydrophila*, and they lay a theoretical foundation for developing strategies to mitigate tetracycline resistance in this aquatic pathogen.

## 2. Materials and Methods

### 2.1. Bacterial Strains, Plasmids and Growth Conditions

*A. hydrophila* NJ-35 and AH823 were cultured in Luria–Bertani Broth (BioDee, Beijing, China) at 28 °C, whereas *Escherichia coli* DH5α was cultured in LB medium at 37 °C. When required, the antibiotic chloramphenicol (Cm) (Macklin, Shanghai, China) was added at a final concentration of 10 μg/mL. The strains and plasmids used in this experiment are listed in Table 1.

### 2.2. Antimicrobial Susceptibility Test

The susceptibility of *A. hydrophila* to tetracycline antibiotics was measured by the disk diffusion method. Briefly, bacterial suspensions were adjusted to a 0.5 McFarland standard (~1.5 × 10^8^ CFU/mL) and then diluted to 1 × 10^7^ CFU/mL. A volume of 100 μL diluted suspensions was spread onto LB agar plates. Antimicrobial paper sheets (Hangzhou Microbial Reagent Co., Ltd., Hangzhou, China), including minocycline (30 μg/piece), tetracycline (30 μg/piece), and doxycycline (30 μg/piece), were affixed on the surface of the plate using the sterile tweezers, followed by incubation at 28 °C for 18 h. The inhibition zone diameters (IZD) were measured, and the results were analyzed according to the guidelines of the Clinical and Laboratory Standards Institute (CLSI) M100 Ed34 (Table S2) [24]. Since no species-specific breakpoints are available for *A. hydrophila*, the Enterobacteriaceae criteria were applied as an approximation (common in aquatic bacteriology). For quality control, the susceptible strain NJ-35 was used as a reference instead of *Escherichia coli*, and its inhibition zones all fell within the susceptible ranges. All assays were conducted with three biological replicates, and each biological sample was further measured in three technical replicates to ensure data reliability.

### 2.3. Minimum Inhibitory Concentration Assay

In accordance with the *Aquaculture Drug Use Guidelines (2025 Nos. 1 and 2)* issued by the Ministry of Agriculture and Rural Affairs of China, doxycycline is a tetracycline antibiotic approved for aquaculture use. Therefore, this drug was selected for subsequent assays to align with the actual practices of aquaculture production. The minimum inhibitory concentration (MIC) of doxycycline against *A. hydrophila* was determined using the broth microdilution method (CLSI M07 Ed12) [24]. Because CLSI does not provide species-specific breakpoints for *A. hydrophila*, the Enterobacteriaceae criteria (susceptible ≤ 4 mg/L, intermediate 8 mg/L, resistant ≥ 16 mg/L) were applied as an approximation. Inoculum was standardized to a 0.5 McFarland turbidity and diluted in LB medium to 1 × 10^6^ CFU/mL. This bacterial suspension was added to 96-well plates containing two-fold serial dilutions of doxycycline (0–128 mg/L). Negative (sterile LB medium) and positive (inoculated LB medium without antibiotic) controls were included, and the susceptible NJ-35 was used as a quality control, with its MIC values consistently ≤ 4 mg/L. After static incubation at 28 °C for 24 h, the MIC was recorded as the lowest concentration preventing visible turbidity, confirmed by OD_600_ readings. Three biological replicates, each with three technical replicates, were performed.

### 2.4. Whole-Genome Resequencing

Whole-genome resequencing was performed on the tetracycline-resistant strain AH823 preserved in our laboratory, using the genome of the tetracycline-susceptible strain NJ-35 (GenBank accession number CP006870.1) as a reference. Purified single colonies were inoculated into 50 mL LB medium and incubated with shaking at 28 °C for 18 h. Bacterial cells were harvested by centrifugation and washed twice, then submitted to Gene De novo Biotech Co. (Guangzhou, China) for whole-genome resequencing on the Illumina X Ten platform (150 bp paired end). Raw reads were filtered using fastp (version 0.20.0) (reads with ≥10% N, > 50% bases Q ≤ 20, or adapters were removed). After filtering, average sequencing depths were ~410× (NJ-35) and ~373× (AH823), with >96% Q20 and >90% Q30 for both samples. Clean reads were aligned to the reference genome using BWA mem (0.7.17). Single nucleotide polymorphisms (SNPs) and insertion deletion (InDel) variants were called with GATK (4.2.6.1) and filtered (QD < 2.0, FS > 60.0, MQ < 40.0, GQ < 20). No de novo assembly was performed. An integrated analysis was conducted by combining the drug resistance phenotypes with the genomic sequencing data to identify candidate mutations responsible for the differential susceptibility to tetracyclines.

### 2.5. Construction of the tetR Antisense RNA-Expressing Strain in A. hydrophila

*tetR* knockdown was carried out following the method described by Darsigny et al. [25], which employs hairpin-forming antisense RNA expressed from a plasmid. Two antisense hairpin sequences targeting the coding regions of *tetR* were designed and synthesized by Sangon Biotech Co., Ltd. (Shanghai, China) (Table 2). The hairpin oligos were annealed, ligated into BamHI/SphI-digested pACYC184, then electroporated into *A. hydrophila* NJ-35. The positive clones (designated AHtetR-as) were cultured in LB medium containing chloramphenicol and identified by detecting the hairpin and 16S rDNA sequences. The RT-qPCR was performed to evaluate the mRNA level of *tetR* in the candidate strains. The primers used for PCR are listed in Table 3. Note that this strategy does not rely on the eukaryotic RNAi machinery (Dicer/RISC), which is absent in *A. hydrophila*. The expressed hairpin RNA is presumed to act through antisense pairing and/or bacterial RNase III mediated degradation [26,27]. However, the exact mechanism remains to be characterized.

An empty vector control strain AH184 was constructed by electroporating the pACYC184 plasmid into *A. hydrophila* NJ-35 to control for non-specific phenotypic effects from plasmid maintenance, antibiotic selection and electroporation. AH184 was used exclusively in the growth curve, antimicrobial susceptibility, and MIC assays. For all other experiments, comparisons were made directly between NJ-35 and AHtetR-as.

### 2.6. Growth Curve

The overnight-cultured *A. hydrophila* was collected, washed and inoculated into the fresh LB liquid medium at a final concentration of 1 × 10^6^ CFU/mL. The cultures were incubated at 28 °C with shaking at 180 rpm for 24 h. The optical densities at 600 nm (OD_600_) were measured every 2 h.

### 2.7. Biofilm Formation Ability

To evaluate the effect of *tetR* knockdown on biofilm formation of *A. hydrophila* upon tetracycline exposure, the crystal violet biofilm assay was performed as previously described [28]. The biofilm formation was determined using the crystal violet staining method, and the absorbance at 570 nm was measured using a full-wavelength microplate reader (Thermo Fisher Scientific, Waltham, MA, USA). The formula calculated the relative biofilm formation capacity:

$$\mathrm{Relative}\;\mathrm{biofilm}\;\mathrm{formation}\;\mathrm{capacity}=\frac{{\mathrm{OD}}_{570}}{{OD}_{600}}$$

### 2.8. Hemolytic Activity

A hemolysis assay was performed following the method described by Long et al. [1] to determine the effect of *tetR* knockdown on the hemolytic capacity of *A. hydrophila*. Briefly, 5 μL of bacterial suspension (1 × 10^6^ CFU/mL) was inoculated onto Columbia blood agar plates and incubated at 28 °C for 24 h. The diameters of the formed hemolytic circles were measured.

### 2.9. Rhodamine B Accumulation Assay

Rhodamine B accumulation was monitored as an indirect indicator of efflux activity according to the method of Wang et al. [29]. In this accumulation assay, a higher intracellular fluorescence intensity indicates greater retention of rhodamine B within the bacterial cells, which generally reflects decreased efflux activity. Conversely, lower fluorescence suggests increased efflux activity. The overnight-cultured *A. hydrophila* was collected, washed and inoculated into the fresh LB liquid medium at a final concentration of 1 × 10^7^ CFU/mL, and incubated with 20 μmol/L rhodamine B at 28 °C under shaking for 2 h. Bacterial pellets were collected by centrifugation at 6000 rpm for 5 min, washed and resuspended in PBS (pH 7.4). Doxycycline was supplemented at 1 MIC, followed by another 2 h of incubation at 28 °C. After centrifugation, 200 μL bacterial suspension was transferred into a sterile black 96-well plate. The fluorescence intensity of rhodamine B was measured at an excitation wavelength of 545 nm and an emission wavelength of 567 nm.

### 2.10. Membrane Potential Assay

Membrane potential (Δ*Ψ*) was monitored using the fluorescent probe 3,3′-dipropylthiadicarbocyanine iodide [DiSC_3_ (5)] according to the method described by Wang et al. [29]. Briefly, bacteria were collected and diluted to 1 × 10^7^ CFU/mL with PBS, then incubated statically at 28 °C. Fluorescence intensity was immediately measured using a SpectraMax Mini Multimode Microplate Reader (Molecular Devices, San Jose, CA, USA) at an excitation/emission wavelength of 622 nm and 670 nm, respectively. At the 10th minute, doxycycline was added to a final concentration of 1/2 MIC (chosen based on preliminary experiments showing consistent trends between 1/2 MIC and MIC, with better stability at sub-MIC), and the fluorescence was recorded at 10 min intervals for 60 min.

### 2.11. Doxycycline Accumulation Analysis

*A. hydrophila* was grown in LB medium to the logarithmic growth phase. Subsequently, cells were incubated with doxycycline (1 MIC) for another 2 h under the same conditions. After incubation, cells were harvested by centrifugation (5000 rpm, 4 °C, 5 min) and washed three times with PBS, then quickly frozen in liquid nitrogen and temporarily stored at −80 °C. The intracellular doxycycline accumulation in *A. hydrophila* was determined by Ultra-high-performance liquid chromatography (Vanquish UHPLC) analysis, coupled with an Orbitrap Exploris™ 480 mass spectrometer (Thermo Fisher Scientific, Waltham, MA, USA). Samples were separated on a HILIC chromatographic column with the column temperature maintained at 25 °C. The flow rate was set at 0.5 mL/min, and the injection volume was 2 μL. The mobile phase consisted of solvent A (water containing 25 mM ammonium acetate and 25 mM ammonia water) and solvent B (acetonitrile). The gradient elution procedure was carried out as follows: 0–0.5 min, maintained at 95% B; 0.5–7 min, linear decrease in B from 95% to 65%; 7–8 min, linear decrease in B from 65% to 40%; 8–9 min, maintained at 40% B; 9–9.1 min, linear increase in B from 40% to 95%; 9.1–12 min, maintained at 95% B. All samples were placed in an automatic sampler at 4 °C throughout the whole detection process. To eliminate systematic signal fluctuation errors, all samples were detected in a random injection order. Quality control (QC) samples were inserted into the sample queue to continuously monitor the stability of the detection system and ensure the reliability and repeatability of metabolomic data. Six biological replicates were prepared for AHtetR-as and NJ-35. Data are shown as peak area per mg of bacterial protein.

### 2.12. Transcriptomic Analysis

Samples were harvested following an identical procedure to the one applied to the doxycycline accumulation sample. The prepared samples were sent to Gene De novo Biotech Co. (Guangzhou, China) for sequencing using the Illumina NovaSeq X Plus platform (Control Software v1.3.1, DRAGEN v4.3). All analyses were performed by OmicsMaster (https://www.omicsmart.com, accessed on 27 April 2026). Each sample was sequenced to an average depth of 11 Gb clean data on the Illumina NovaSeq X Plus platform, with a Q30 quality score of ≥96%. Gene expression levels were quantified using the Fragments Per Kilobase of transcript per Million mapped reads (FPKM) method. Differentially expressed genes were screened at the threshold of fold change ≥ 2 and FDR < 0.05. The methods and procedures for transcriptome bioinformatics analysis are shown in Table S1 [30,31,32].

### 2.13. RT-qPCR

The *tetR* knockdown efficiency and the validity of the transcriptomic data were verified by RT-qPCR. In brief, total RNA was extracted from *A. hydrophila* using RNAiso Plus (TaKaRa, Japan) according to the manufacturer’s instructions. The cDNA was synthesized using a reverse transcription kit (Vazyme, Nanjing, China). qPCR was performed on a CFX96 Touch Real-time PCR System (Bio-Rad, Hercules, CA, USA). The 16S rRNA gene was selected as the internal reference after confirming its stable expression across all samples (coefficient of variation of Ct values < 5%). Amplification efficiencies ranged from 90% to 110% with R^2^ values > 0.98. Melting curve analysis was performed for each reaction (60 °C to 95 °C with 0.5 °C increments) to confirm amplification specificity and the absence of primer dimers. Relative expression levels of the target genes were calculated using the 2^−ΔΔCt^ method. All RT-qPCR primer pairs are listed in Table 4.

### 2.14. Statistical Analysis

Unless otherwise specified, all experimental data analyses in this study were conducted with three parallel samples per group and independently replicated three times. All data are presented as mean ± SD. Statistical analysis was performed using GraphPad Prism 8 (San Diego, CA, USA). An unpaired two-tailed Student’s *t*-test (normally distributed data) was used between two groups, and one-way ANOVA followed by Tukey’s HSD test was conducted among multiple groups. A value of *p* < 0.05 was considered statistically significant, and *p* < 0.01 indicated an extremely substantial difference.

## 3. Results

### 3.1. Analysis of Tetracycline Resistance-Associated Genes

**Figure 1:**
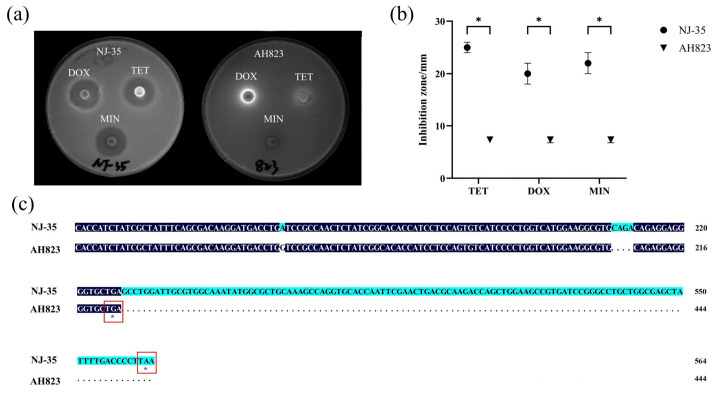
Testing of *A. hydrophila*’s susceptibility to tetracycline antibiotics. (**a**) The inhibition zone can be seen as a clear circle caused by the antimicrobial disk. (**b**) The inhibition zone diameters (IZD) were measured to evaluate the susceptibility of *A. hydrophila* to doxycycline (DOX), minocycline (MIN) and tetracycline (TET). The values are presented as mean ± SD and the significance was determined by a *t*-test (* *p* < 0.05). (**c**) Alignment of *tetR* from NJ-35 and AH823. The mutations in AH823 create a premature stop codon (TGA, red box). NJ-35 carries the normal termination codon (TAA, red box).

The results of antimicrobial susceptibility testing of tetracycline antibiotics against *A. hydrophila* demonstrated that NJ-35 was sensitive to tetracycline, while AH823 exhibited high-level resistance to tetracycline antibiotics (Figure 1a,b).

Compared to NJ-35, AH823 contained 43,131 SNPs (99.5% homozygous) and 363 indels, indicating a highly homozygous genetic background. Among the tetracycline resistance-associated genes (*tetR*, *tet*(A), *rnd*), only *tetR* carried base deletions (supported by depth > 300× and a homozygous background). As shown in Figure 1c, *tetR* in AH823 harbored an A → G point mutation at position 148 and a 4-nucleotide CAGA deletion spanning positions 212 to 215, which likely causes premature translational termination.

### 3.2. Construction of AHtetR-as

**Figure 2:**
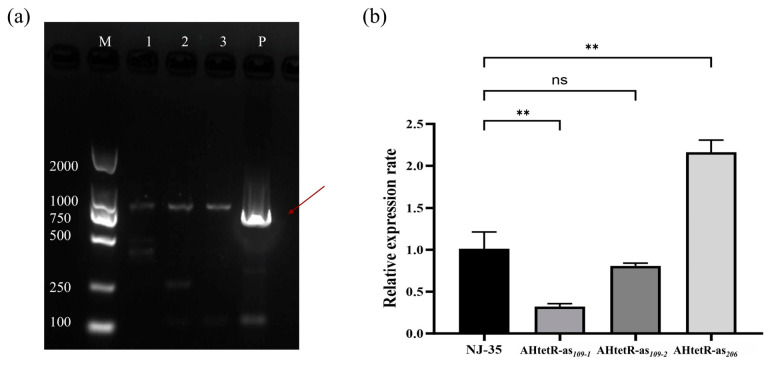
Identification of AHtetR-as derived from *A. hydrophila* NJ-35. (**a**) PCR verification with pACYC184 vector-specific primers. M, Marker DL2000; 1, AHtetR-as*_109-1_*; 2, AHtetR-as*_109-2_*; 3, AHtetR-as*_206_*; P, pACYC184 plasmid. The red arrow indicates the PCR amplification product (approximately 750 bp) (**b**) The transcriptional level of *tetR* in three candidates. The values are presented as mean ± SD and the significance was determined by ANOVA (** *p* < 0.01; ns *p* > 0.05).

A plasmid expressing an antisense RNA targeting *tetR* (*tetR*-asRNA) was electroporated into NJ-35, yielding three candidate *tetR*-knockdown strains, designated as AHtetR-as*_109-1_*, AHtetR-as*_109-2_*, and AHtetR-as*_206_*. Subsequently, 16S rDNA sequencing confirmed these candidates as *A. hydrophila*, and PCR amplification verified the presence of the *tetR*-asRNA plasmid in all three strains (Figure 2a).

The efficiency of *tetR* knockdown was detected by RT-qPCR. The results showed that the transcriptional level of *tetR* in AHtetR-as*_109-1_* was significantly reduced by 70% compared to that of NJ-35, which exhibited the highest knockdown efficiency among the three candidates. Consequently, this strain was designated AHtetR-as and selected as the experimental strain for all subsequent assays (Figure 2b).

### 3.3. Antimicrobial Susceptibility Test Analysis

**Figure 3:**
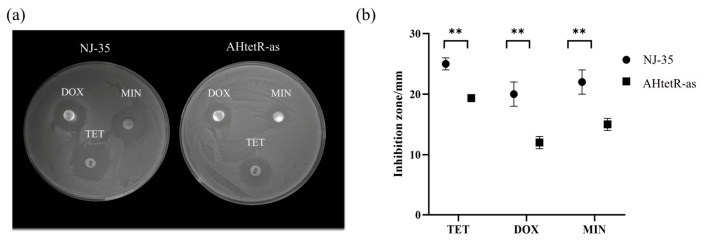
Effects of antisense-mediated knockdown of *tetR* on the susceptibility of *A. hydrophila* to tetracycline antibiotics. (**a**) The drug sensitivity test was determined by the disk diffusion method. (**b**) Quantification of drug susceptibility tests based on inhibition zone diameter. The values are presented as mean ± SD and the significance was determined by a *t*-test (** *p* < 0.01).

As illustrated in Figure 3, AHtetR-as displayed a significant increase in resistance to tetracycline, doxycycline and minocycline relative to the wild-type strain NJ-35. Specifically, the doxycycline MIC was 1 mg/L for the wild-type strain NJ-35, 4 mg/L for the empty vector control AH184, and 16 mg/L for the *tetR*-knockdown strain AHtetR-as. AH184 carries pACYC184, which contains a tetracycline resistance gene, and that this gene was disrupted in the antisense construct used for AHtetR-as. Overall, AHtetR-as exhibited a 16-fold higher MIC compared to NJ-35.

### 3.4. Growth Curve Analysis

**Figure 4:**
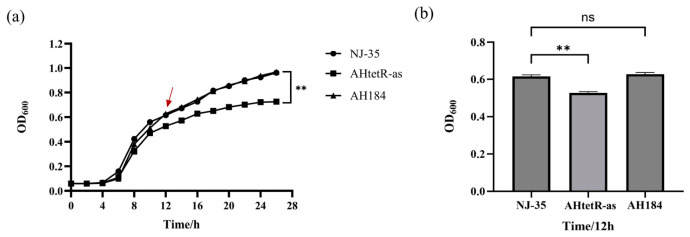
Growth curve of *A. hydrophila*. (**a**) Growth curves of NJ-35, AH184 and AHtetR-as. Statistically significant differences between AHtetR-as and NJ-35 began at the 12 h time point (as indicated by arrows). Statistical comparisons were performed at all measured time points (every 2 h). (**b**) OD_600_ of the three strains at 12 h. The values are presented as mean ± SD and the significance was determined by a *t*-test (** *p* < 0.01).

As shown in Figure 4a,b, the growth rate of AH184 was consistent with that of NJ-35, with no observable effect under the tested conditions. Furthermore, the growth curves of AHtetR-as and NJ-35 revealed that after the lag phase, AHtetR-as grew slightly slower than NJ-35, with statistically significant differences beginning at the 12 h time point and continuing over the subsequent hours (indicated by an arrow). Statistical comparisons were performed at all measured time points (every 2 h).

### 3.5. Effects of tetR Knockdown on Virulence Phenotypes of A. hydrophila

To determine whether *tetR* knockdown affects virulence-associated phenotypes, we compared the wild type strain NJ 35 and the knockdown strain AHtetR-as in biofilm formation and hemolytic activity assays.

Biofilm formation was measured by crystal violet staining after 24 h at 28 °C. No significant difference was observed between the two strains (*p* > 0.05) (Figure S1a).

Hemolytic activity was determined by measuring the diameter of the hemolytic zone formed by bacteria on blood agar plates. Both strains exhibited *β*-hemolysis, with no significant difference in hemolytic zone diameter (*p* > 0.05) (Figure S1b).

Thus, *tetR* knockdown did not significantly alter biofilm formation or hemolytic activity under the tested conditions.

### 3.6. Effects of tetR Knockdown on the Efflux Pump of A. hydrophila

**Figure 5:**
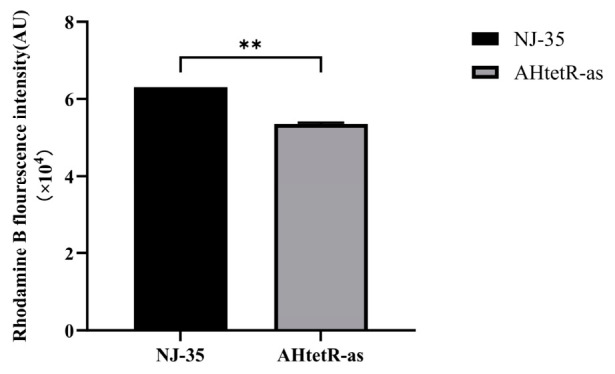
Effects of *tetR* knockdown on efflux pump activity of *A. hydrophila* upon doxycycline exposure, measured with the fluorescence dye rhodamine B. The values are presented as mean ± SD and the significance was determined by a *t*-test (** *p* < 0.01).

As depicted in Figure 5, the intracellular fluorescence intensity of NJ-35 was higher than that of AHtetR-as. Lower intracellular fluorescence indicates increased efflux-associated substrate removal.

### 3.7. Effects of tetR Knockdown on Proton Motive Force of A. hydrophila

**Figure 6:**
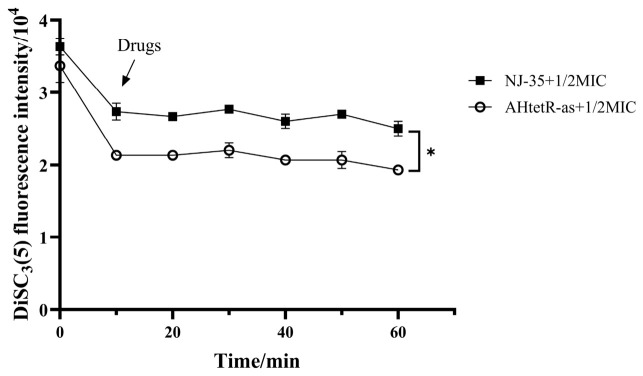
Membrane potential changes in *A. hydrophila* upon doxycycline exposure, probed by potentiometric fluorophore DiSC_3_(5). DiSC_3_(5) dye was injected at 10 min. The values are presented as mean ± SD and the significance was determined by a *t*-test (* *p* < 0.05).

To explore the effect of *tetR* knockdown on Δ*Ψ*, we monitored fluorescence changes in the membrane potential-sensitive dye DiSC_3_(5). As depicted in Figure 6, addition of doxycycline at 1/2MIC decreased DiSC_3_(5) fluorescence, and lower fluorescence indicates dye efflux due to membrane hyperpolarization (an indicator of increased Δ*Ψ*). The fluorescence decrease was greater in AHtetR-as than in NJ-35.

### 3.8. Effects of tetR Knockdown on Intracellular Doxycycline Accumulation of A. hydrophila

**Figure 7:**
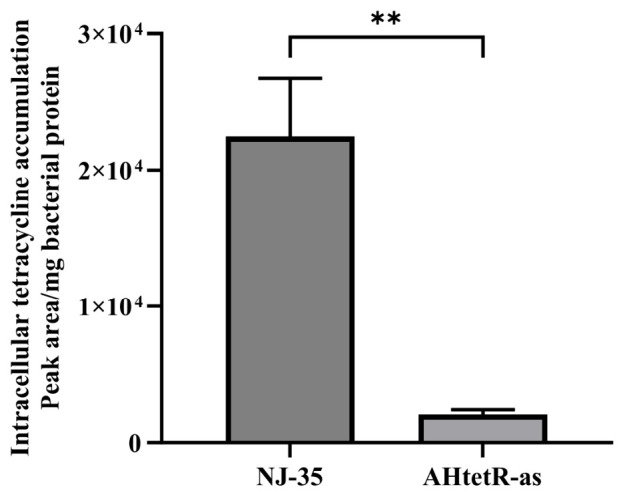
Intracellular accumulation of doxycycline metabolites in *A. hydrophila* treated with doxycycline. The values are presented as mean ± SD and the significance was determined by a *t*-test (** *p* < 0.01).

As shown in Figure 7, AHtetR-as exhibited significantly diminished intracellular accumulation of doxycycline metabolites compared to NJ-35. This prominent difference is consistent with increased efflux-associated activity.

### 3.9. Transcriptome Analysis

**Figure 8:**
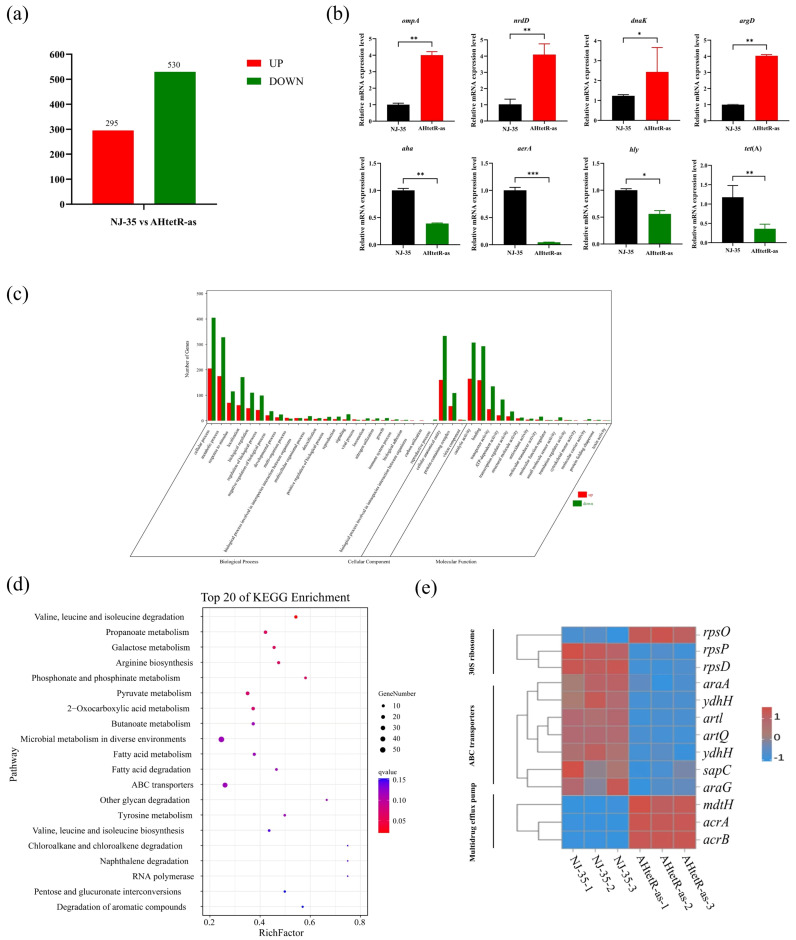
Transcriptome analysis between the knockdown strain AHtetR-as and the wild-type strain NJ-35. (**a**) Number of DEGs. (**b**) RT-qPCR verification. (* *p* < 0.05; ** *p* < 0.01, *** *p* < 0.001) (**c**) GO functional enrichment. (**d**) The Top 20 of KEGG enrichment. The larger the circle in the KEGG map, the more differential gene enrichment, and the redder the color, the higher the significance. (**e**) DEGs associated with the 30S ribosome, ABC transporters, and multidrug efflux pumps.

The comparison of AHtetR-as to NJ-35 identified a total of 825 significantly differentially expressed genes (DEGs), among which 295 DEGs were upregulated and 530 DEGs were downregulated (Figure 8a). Based on the GO database, these DEGs can be classified into three categories: biological process, cellular component, and molecular function (Figure 8c). According to the KEGG database, the metabolism category contained the largest number of DEGs. Notably, pathways including ABC transporters, valine–leucine–isoleucine degradation and arginine biosynthesis were significantly enriched (Figure 8d).

Transcriptional regulation revealed by RNA-seq data was verified via RT-qPCR, for which a total of eight genes were selected. The RT-qPCR result displayed a good correlation as the transcription profile of RNA-seq data (Figure 8b), supporting the expression trends observed in the transcriptomic data.

To further explore the potential effect of *tetR* knockdown on selected drug resistance-associated genes in *A. hydrophila*, we compared the transcriptional levels of genes related to the 30S ribosomal subunit, ABC transporters, and multidrug efflux pumps between AHtetR-as and NJ-35 (Figure 8e). Genes encoding ABC transporters showed a global downregulation trend. Conversely, multidrug efflux pump genes were significantly upregulated.

## 4. Discussion

*A. hydrophila* is widely recognized as a major aquatic pathogen, with its widespread prevalence imposing a considerable threat on the aquaculture industry [33,34]. Notably, this pathogen can trigger high-mortality bacterial outbreaks in Hybrid Sturgeon (*Huso dauricus* ♀ × *A. schrenckii* ♂), resulting in severe economic losses and social impacts across China, Europe, and the United States [35,36]. A growing concern, moreover, is the emergence of AMR in *A. hydrophila* driven by the overuse and misuse of antibiotics in aquaculture. An early study has reported that a collection of 313 *Aeromonas* isolated from rainbow trout and their aquaculture environments in Denmark exhibited predominant resistance to oxytetracycline (OTC, one of the first developed natural tetracyclines). The resistance rate to OTC was as high as 69% [37]. And more recently, Thaotumpitak et al. [38] found that 53.4% of 15 *A. hydrophila* among 278 total bacterial isolates were resistant to tetracycline antibiotics. These findings underscore the urgency of addressing tetracycline resistance in *A. hydrophila* for sustainable aquaculture disease control.

A clinical isolate of *A. hydrophila* AH823 exhibiting high-level doxycycline resistance was isolated from diseased *Alosa sapidissima*. Genomic comparison with the susceptible strain NJ-35 revealed *tetR* mutations in AH823. To explore whether *tetR* disruption contributes to tetracycline resistance, we constructed a plasmid-based antisense RNA-expressing strain targeting *tetR* in NJ-35, designated AHtetR-as. We recognize that this antisense approach may lead to only partial and variable knockdown. The empty vector control strain AH184 showed a 4-fold higher doxycycline MIC than NJ-35, which can be attributed to the tetracycline resistance marker on the pACYC184 backbone. Because this marker was disrupted in the antisense construct used for AHtetR-as, the additional 4-fold MIC increase (from 4 to 16 mg/L) in this strain likely results from *tetR* knockdown rather than from vector effects. We acknowledge the lack of complementation experiments, and the absence of direct measurement. A major limitation is the lack of complementation experiments, leaving the causal relationship between *tetR* and resistance unproven. In addition, we did not directly measure antisense RNA expression or plasmid stability, nor did we systematically assess off-target effects. Therefore, alternative explanations such as plasmid burden, antisense toxicity, and off-target effects cannot be entirely excluded.

Biological characterization showed that AHtetR-as displayed a slower growth rate than NJ-35, but there were no significant differences in biofilm formation or hemolytic activity, suggesting that *tetR* may be involved in bacterial growth. These findings indicate that *tetR* contributes to tetracycline resistance in *A. hydrophila*, though the underlying mechanism requires further investigation.

Efflux pumps mediate bacterial drug resistance by expelling antibiotics to reduce intracellular accumulation [39]. The energy for tetracycline efflux is predominantly derived from PMF, comprising Δ*Ψ* and the transmembrane proton gradient (ΔpH) [40]. In the current study, *tetR* knockdown led to reduced rhodamine B accumulation, which is consistent with but does not directly prove increased efflux of this RND-type substrate. It also led to reduced doxycycline accumulation, similarly suggestive of increased doxycycline efflux. These changes were accompanied by an elevated Δ*Ψ*, with the limitations noted below. Taken together, these observations are suggestive of a possible enhancement in PMF-dependent efflux activity under *tetR* knockdown conditions, although direct evidence is lacking. However, direct quantification of PMF was not performed, and no protonophore (e.g., CCCP) was used as a positive control for membrane depolarization. Therefore, the elevated Δ*Ψ* alone does not constitute direct evidence of increased PMF-driven efflux. While the elevated Δ*Ψ* is consistent with the reduced accumulation of both substrates, alternative interpretations—such as changes in membrane permeability or nonspecific probe retention—cannot be excluded. Direct measurement of both PMF components (Δ*Ψ* and ΔpH) and real-time efflux rates, along with CCCP controls, is required to resolve the underlying mechanisms.

The tetracycline-specific efflux pump Tet(A) belongs to the MFS family, and is typically repressed by TetR in the classical Tn10-encoded system of *E. coli* [41]. In our *A. hydrophila* isolate, TetR was initially predicted to repress *tet*(A) expression based on homology with this system, although *tet* operon regulation is known to vary across species and genetic contexts [42]. Contrary to the classical model, *tetR* knockdown in NJ-35 led to marked downregulation of *tet*(A) mRNA, which was inconsistent with our expectations. This suggests that the classical TetR-TetA negative regulation may not apply in this genetic background. The exact mechanism remains unknown and requires experimental dissection.

Transcriptomic analyses revealed upregulation of multidrug efflux pump genes (*arcA*, *arcB* and *mdtH*) and downregulation of ABC transporter-related genes in the knockdown strain. Multidrug efflux pumps are known drivers of OTC resistance in *A. hydrophila* [37]. ABC transporters are primary ATP-driven systems, and their reduced expression may reflect energy reallocation rather than a direct role in resistance, although functional assays are needed to clarify this. In *E. coli*, Tet(A) resistance depends on the AcrAB-TolC efflux pump [43], but regulatory networks in Aeromonas are not necessarily conserved. We therefore use this comparison only as a reference, not as direct evidence. We hypothesize that in *A. hydrophila*, *tetR* knockdown upregulates multidrug efflux pump indirectly rather than via direct promoter activation, a possibility that requires experimental testing. This upregulation could contribute to a broad-spectrum resistance network, potentially repressing the tetracycline-specific efflux pump.

Tetracyclines inhibit protein synthesis by binding to the 30S ribosomal subunit [9]. In our study, genes associated with the 30S ribosomal subunit exhibited differential expression. The core assembly factor gene *rpsO* was upregulated, while the structural protein genes *rpsD* and *rpsP* were downregulated. Changes in the expression of several 30S ribosomal protein genes suggest that ribosome-associated processes may be altered following *tetR* knockdown.

Taken together, the acquired tetracycline resistance following *tetR* knockdown in *A hydrophila* appears to involve a coordinated regulatory network based on our transcriptomic data. On the one hand, *tetR* knockdown is associated with upregulation of broad-spectrum multidrug efflux pumps and altered energy metabolism, which could accelerate tetracycline efflux and reduce its intracellular accumulation. On the other hand, it is associated with altered expression of 30S ribosomal genes. A plausible hypothesis is that such changes could disrupt ribosome integrity and thereby potentially reduce tetracycline binding affinity. These interpretations remain correlative and require direct experimental validation. Future multi-omics analysis, such as transcriptomics combined with metabolomics, could help further elucidate the energy pathways and regulatory network involved.

## 5. Conclusions

This study successfully constructed a stable *tetR* antisense RNA-expressing strain AHtetR-as from a tetracycline-susceptible *A. hydrophila* isolate. *tetR* knockdown conferred increased tetracycline resistance without affecting hemolytic activity or biofilm formation, suggesting that *tetR* modulates drug resistance independently of these virulence traits. Transcriptomic analysis revealed altered expression of broad-spectrum multidrug efflux pump genes and 30S ribosomal subunit genes, pointing to two possible mechanisms associated with the resistant phenotype. However, direct evidence for efflux activity or ribosomal dysfunction is lacking. These mechanisms remain correlative and require functional verification. Contrary to the classical model, *tetR* knockdown in NJ-35 led to marked downregulation of *tet*(A) mRNA, which was inconsistent with our expectations. This suggests that the classical TetR-TetA negative regulation may not apply in this genetic background. The exact mechanism remains unknown and requires experimental dissection. In conclusion, *tetR* appears to play a role in acquired tetracycline resistance in *A. hydrophila*, but the regulatory network is not yet fully resolved. This study’s limitations include the correlative nature of transcriptomic data, the use of shRNA knockdown (which does not fully replicate native regulation), and the lack of complementation experiments. Thus, these findings are preliminary and require direct functional assays and genetic complementation.

## Figures and Tables

**Table 1:** Experimental strains and plasmids.

Plasmids or Strains	Genotype or Phenotype	Source
Plasmids		
pACYC184	(Cm^r^ Tc^r^)	Miaolingbio (Wuhan, China)
pACYC184-*tetR*	The recombinant plasmid pACYC184-*tetR* was transformed into *E. coli* DH5α	This study
Strains		
NJ-35	Wild-type strain of *A. hydrophila*	Provided by Prof. Yongjie Liu
AH823	*A hydrophila*, isolated from diseased *Alosa sapidissima* (TET^r^, DOX^r^, MIN^r^)	Collected in our laboratory
AH184	An empty vector control strain of *A. hydrophila* NJ-35 containing pACYC184 empty vector (Cm^r^)	This study
DH5α	F^−^, φ80d*lacZ*ΔM15, Δ(*lacZYA-argF*) U169, *deoR*, *recA1 endA1*, *hsdR17* (rK^−^ mK^+^), *phoA*, *supE44*, λ^−^, thi-1, *gyrA96*, *relA1*	TaKaRa (Kusatsu, Japan)
AHtetR-as		This study
AHtetR-as*_109-1_*	*tetR* antisense RNA-expressing candidate strains of *A hydrophila* (Cm^r^)	
AHtetR-as*_109-2_*		
AHtetR-as*_206_*		

**Table 2:** Antisense hairpin sequence for stable gene knockdown.

Primer	Sequence (5′-3′)
i206F	GATCCTGCAGACAGAGGAGGTATCGTTCAAGAGACGATACCTCCTCTGTCTGCATTTTTTGCATG
i206R	CAAAAAATGCAGACAGAGGAGGTATCGTCTCTTGAACGATACCTCCTCTGTCTGCAG
i109F	GATCCGGCACCATCTATCGCTATTTCTCAAGAGGAAATAGCGATAGATGGTGCCTTTTTTGCATG
i109R	CAAAAAAGGCACCATCTATCGCTATTTCCTCTTGAGAAATAGCGATAGATGGTGCCG

**Table 3:** Primers used for PCR in the experiment.

Primer	Sequence (5′-3′)	Purpose
27F	AGAGTTTGATCCTGGCTTAG	Identification of 16S rDNA
1492R	ACGGCTACCTTGTTACGACTT	
Tcr-F	GAAGTCATGCGCCGGTTAAG	Detection of shRNA in pACYC184
pBRrevBam-R	GGTGATGTCGGCGATATAGG	

**Table 4:** Primers for RT-qPCR.

Gene	Primer Sequence	Gene	Primer Sequence
16S rRNA-F	TAATACCGCATACGCCCTAC	*ompA* F	TGGATCTGCAAGCTCGTTAC
16S rRNA-R	GGACCGTGTCTCAGTTCCAG	*ompA* R	CTACGTAGGAAGTGCGGAAC
91F	AATTTCGGCGTTTGTGGCTC	*aha* F	AAGCCGTCAAGGTTACTGAC
91R	AGCGGGGACGTTTCATACTG	*aha* R	GTCACCAGTGTTGTTGGTCT
*tet*(A) F	TTGCAGCCTCAACAATTGCC	*argD* F	GTGCCGAAGCCAACGAA
*tet*(A) R	AAACGGTGCCTGTACCGAAA	*argD* R	CCGACGCTGACGGTAAAG
*aerA* F	AGGAGATGTCAGCCTTGTAG	*nrdD* F	GGCGATCCCAACTACGACATG
*aerA* R	TTACGATACCGCCACCAACT	*nrdD* R	CGTAGGCACCGAGGAAAGAG
*hly* F	TCTACCTCAACGTCAACCGC	*act* F	CGAATTCGTTCATCACCGGC
*hly* R	TCCGCACTATCTTGGCATCC	*act* R	ACTACAGCCTGTTCAGCGAC
*dnaK* F	GCCAAGCCATCACCAA		
*dnaK* R	TTGCCTTTGACTTCTACCC		

## Data Availability

The original contributions presented in this study are included in the article/Supplementary Materials. Further inquiries can be directed to the corresponding authors.

## Supplementary Materials

The following supporting information can be downloaded at: https://www.mdpi.com/article/10.3390/vetsci13060577/s1, Figure S1: Effects of *tetR* knockdown on virulence phenotypes of *A. hydrophila*. (a) Biofilm formation ability. (b) Hemolytic activity. The values are presented as mean ± SD and the significance was determined by a *t*-test (* *p* < 0.05; ns *p* > 0.05); Table S1: Prokaryotic transcriptome data analysis method; Table S2: Classification standards for bacterial susceptibility to tetracycline, doxycycline and minocycline.
