# Supplementary material for: Functional Characterization of tetR in Tetracycline Resistance of Aeromonas hydrophila
Source: Vet Sci. 2026 Jun 12;13(6):577. doi: 10.3390/vetsci13060577 (PMC13308043; doi:10.3390/vetsci13060577)
Supplement: Supplementary file 1 [file vetsci-13-00577-s001.zip › Supplement Material.pdf]

Supplement Materials

Supplement Figure

Figure S1 Effects of *tetR* knockdown on virulence phenotypes of *A. hydrophila*. (a) Biofilm formation ability. (b) Hemolytic activity. The values were presented as mean ± SD and the significance was determined by *t*-test (*$P < 0.05$; ns $P > 0.05$).

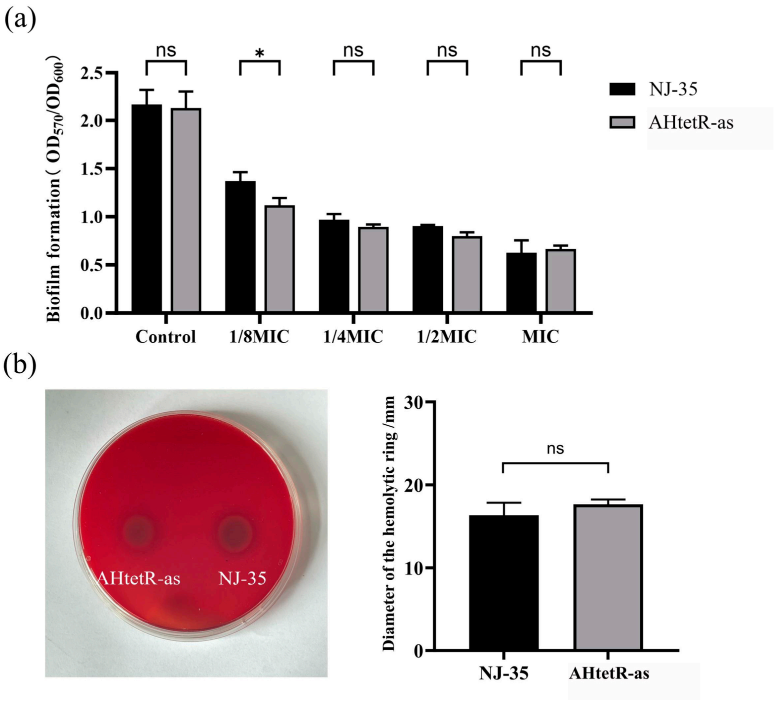

Supplement Table

Table S1 Prokaryotic transcriptome data analysis method

Category	Analysis Content	Tools/Databases Used	Version and References
Data filtering	Filtering of raw data from Illumina platform	FASTP	(version 0.20.0) [1]
	Clean reads were mapped to the reference genome and reads mapped to ribosome RNA were removed	Bowtie2	(version 2.2.8) [2]
	Calculated gene expression	RSEM	(version:1.2.19) [3]
Analysis of samples correlation and principle component	The correlation coefficient among samples was calculated.	R package psych	(version 1.8.4) [4]
Analysis of differentially expressed genes	Identify differentially expressed genes (DEGs) among samples with fold change ⩾ 2 and FDR< 0.05. DEGs were used to analyze GO and KEGG, and FDR < 0.05 was used as threshold.	edgeR package	(version 3.12.1) [5]
sRNA analysis	Predict sRNA and the removal of sequences less than 50 bp.	Rockhopper	(version 2.0.3) [6]
	Screen candidate sRNAs	sRNAMap database, Rfam database	(version 2009) (version13) [7,8]
	Predict secondary structures of sRNA	Vienna RNA packages	(version 2.3.5) [9]
Transcript structure analysis	Predict new transcripts	Rockhopper	(version 2.0.3)
	Verify candidate cis-NATs and cis-NATs with minimum free	RNAplex	(version 0.2) [10]

	energy more than -30 kcal/mol were removed.		
	Predict Shine-Dalgarno sequence and set the parameter of ribosome region length 20 bp.	RBSfinder	[11]
	Identify ρ-independent terminator	TransTermHP	(version 2.09) [12]
Trend analysis	Gene expression pattern analysis is used to cluster genes of similar expression patterns for multiple samples (at least 3 in a specific time point, space, or treatment dose). To evaluate the expression pattern of DEGs, the expression data of each sample was normalized and then clustered.	ShortTime-series Expression Miner (STEM)	(version 1.3.11) [13]
Gene Set Enrichment Analysis (GSEA)	Perform the gene set enrichment analysis in order to identify a set of genes in specific GO terms\KEGG Pathways which show significant differences between two groups.	GSEA, MSigDB	[14]

Table S2 Classification standards for bacterial susceptibility to tetracycline, doxycycline and minocycline

Antibiotic	Resistant (mm)	Intermediate (mm)	Susceptible (mm)
Tetracycline	≤ 14	15–18	≥ 19
Doxycycline	≤ 10	11–13	≥ 14
Minocycline	≤ 13	14–18	≥ 19

Note: Species-specific breakpoints for *A. hydrophila* were referred to the Enterobacteriaceae criteria according to CLSI M100 Ed34 (a common practice in aquatic bacteriology).

Reference

1. Chen, S.; Zhou, Y.; Chen, Y.; Gu, J. fastp: an ultra-fast all-in-one FASTQ preprocessor. *Bioinformatics* **2018**, *34*, i884–90. doi:10.1093/bioinformatics/bty560.
2. Langmead, B.; Salzberg, S.L. Fast gapped-read alignment with bowtie 2. *Nat Methods* **2012**, *9*, 357–9. doi:10.1038/nmeth.1923.
3. Li, B.; Dewey, C.N. RSEM: accurate transcript quantification from RNA-seq data with or without a reference genome. *BMC Bioinf.* **2011**, *12*, 323. doi:10.1186/1471-2105-12-323.
4. Revelle, W. psych: procedures for psychological, psychometric, and personality research. **2007**, 2.5.6. Available at: https://CRAN.R-project.org/package=psych. Accessed January 8, 2026.
5. Robinson, M.D.; McCarthy, D.J.; Smyth, G.K. edgeR : A a bioconductor package for differential expression analysis of digital gene expression data. *Bioinformatics* **2010**, *26*, 139–40. doi:10.1093/bioinformatics/btp616.
6. McClure, R.; Balasubramanian, D.; Sun, Y.; Bobrovskyy, M.; Sumby, P.; Genco, C.A.; Vanderpool, C.K.; Tjaden, B. Computational analysis of bacterial RNA-seq data. *Nucleic Acids Res*. **2013**, *41*, e140–e140. doi:10.1093/nar/gkt444.
7. Huang, H.Y.; Chang, H.Y.; Chou, C.H.; Tseng, C.P.; Ho, S.Y.; Yang, C.D.; Ju, Y.W.; Huang, H.D. sRNAMap: genomic maps for small non-coding RNAs, their regulators and their targets in microbial genomes. *Nucleic Acids Res*. **2009**, *37*, D150–4. doi:10.1093/nar/gkn852.
8. Kalvari, I.; Argasinska, J.; Quinones-Olvera, N.; Nawrocki, E.P.; Rivas, E.; Eddy, S.R.; Bateman, A.; Finn, R.D.; Petrov, A.I. Rfam 13.0: shifting to a genome-centric resource for non-coding RNA families. *Nucleic Acids Res*. **2018**, *46*, D335–42. doi:10.1093/nar/gkx1038.

9. Gruber, A.R.; Lorenz, R.; Bernhart, S.H.; Neubock, R.; Hofacker, I.L. The Vienna RNA websuite. *Nucleic Acids Res*. **2008**, *36*, W70–4. doi:10.1093/nar/gkn188.
10. Tafer, H.; Hofacker, I.L. RNAplex: a fast tool for RNA – RNA interaction search. *Bioinformatics* **2008**, *24*, 2657–63. doi:10.1093/bioinformatics/btn193.
11. Suzek, B.E.; Ermolaeva, M.D.; Schreiber, M.; Salzberg, S.L. A probabilistic method for identifying start codons in bacterial genomes. *Bioinformatics* **2001**, *17*, 1123 – 30. doi:10.1093/bioinformatics/17.12.1123.
12. Kingsford, C.L.; Ayanbule, K.; Salzberg, S.L. Rapid, accurate, computational discovery of rho-independent transcription terminators illuminates their relationship to DNA uptake. *Genome Biol*. **2007**, *8*, R22. doi:10.1186/gb-2007-8-2-r22.
13. Ernst, J.; Bar-Joseph, Z. STEM: a tool for the analysis of short time series gene expression data. *BMC Bioinf.* **2006**, *7*, 191. doi:10.1186/1471-2105-7-191.
14. Subramanian, A.; Tamayo, P.; Mootha, V.K.; Mukherjee, S.; Ebert, B.L.; Gillette, M.A.; Paulovich, A.; Pomeroy, S.L.; Golub, T.R.; Lander, E.S.; et al. Gene set enrichment analysis: a knowledge-based approach for interpreting genome-wide expression profiles. *Proc Natl. Acad. Sci. U.S.A.* **2005**, *102*, 15545–50. doi:10.1073/pnas.0506580102.
