# Supplementary figures and images for: Functional Characterization of tetR in Tetracycline Resistance of Aeromonas hydrophila
Source: Vet Sci. 2026 Jun 12;13(6):577. doi: 10.3390/vetsci13060577 (PMC13308043; doi:10.3390/vetsci13060577)

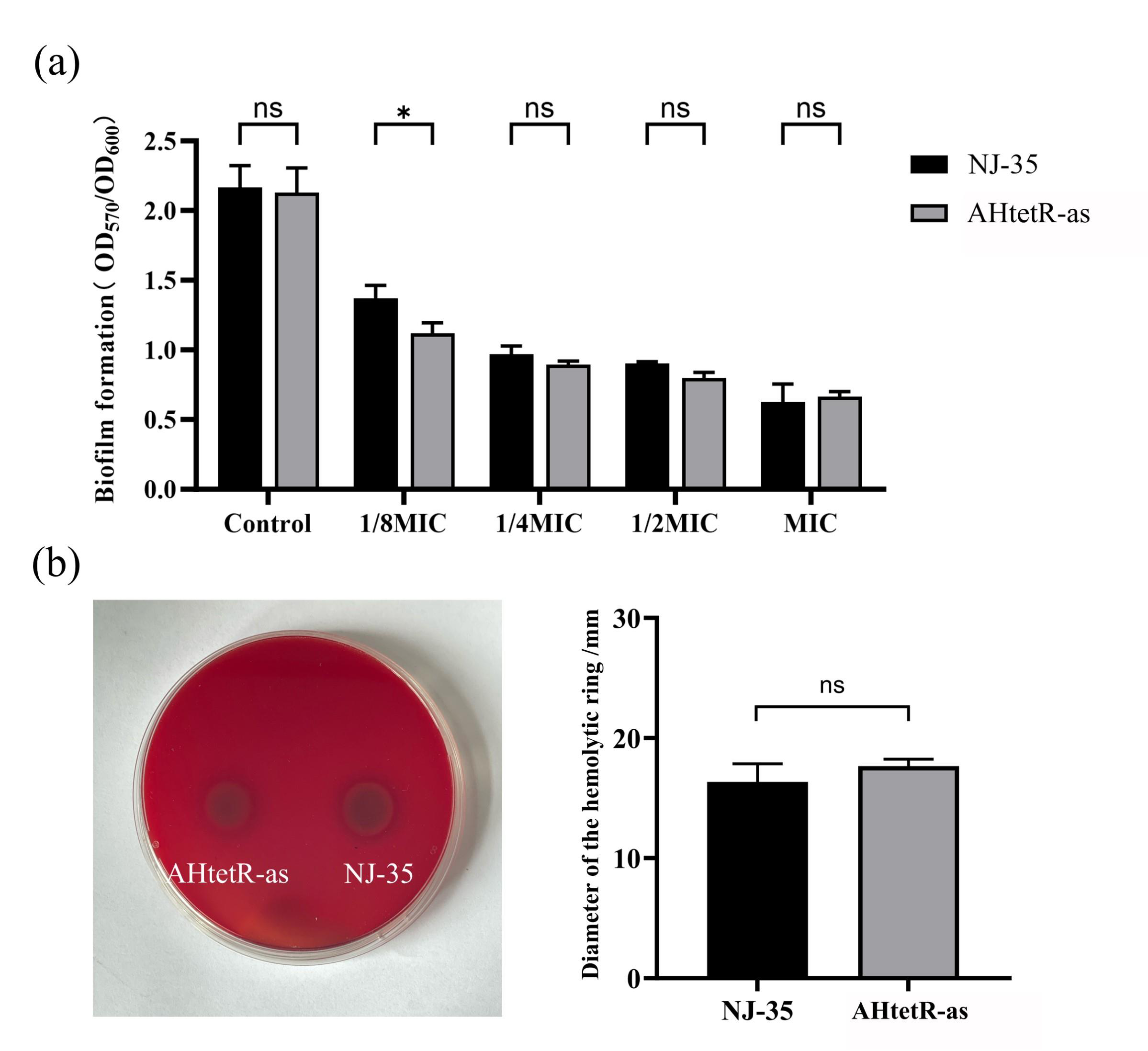

Supplement: Supplementary file 1 [file vetsci-13-00577-s001.zip › Figure S1.tif]
